# Cost-effectiveness of paclitaxel plus cisplatin in advanced non-small-cell lung cancer

**DOI:** 10.1038/sj.bjc.6690426

**Published:** 1999-05

**Authors:** C C Earle, W K Evans

**Affiliations:** Ottawa Regional Cancer Centre, University of Ottawa and Cancer Care Ontario, Ottawa, Ontario, K1H 8L6, Canada

**Keywords:** non-small cell lung cancer, costs, chemotherapy, paclitaxel, cisplatin

## Abstract

The aim of this study was to assess the cost-effectiveness of combination chemotherapy with paclitaxel/cisplatin, compared with standard etoposide/cisplatin in patients with advanced non-small cell lung cancer (NSCLC). We obtained the primary survival and resource utilization data from a large three-arm randomized trial comparing: paclitaxel 135 mg m^−2^ by 24-h intravenous (i.v.) infusion + cisplatin; paclitaxel 250 mg m^−2^ by 24-h i.v. infusion + cisplatin + granulocyte colony-stimulating factor (G-CSF); and standard etoposide/cisplatin in patients with stage IIIb or IV NSCLC. We also modelled the regimens with paclitaxel 135 mg m^−2^ + cisplatin administered as an outpatient by 3-h infusion, as clinical data suggest that this is equivalent to 24-h infusion. We collected costing data from the Ottawa Regional Cancer Centre and applied it to the resources consumed in the randomized trial. We integrated these data into the Statistics Canada POpulation HEalth Model (POHEM), which generated hypothetical cohorts of patients treated with each regimen. The POHEM model assigned diagnostic work-up, treatment, disease progression and survival characteristics to each individual in these cohorts and tabulated the costs associated with each. We did sensitivity analyses around the costs of chemotherapy and its administration, and the survival differences between the two regimens. All costs are in 1997 Canadian dollars ($1.00 Canadian ~ £0.39 sterling). The perspective is that of the Canadian health care system. In the trial, the two paclitaxel-containing arms had almost identical survival curves with a median survival of 9.7 months compared with 7.4 months for etoposide/cisplatin. As administered in the trial, paclitaxel/cisplatin cost $76 370 per life-year gained (LYG) and paclitaxel/cisplatin/G-CSF $138 578 per LYG relative to etoposide/cisplatin. However, when modelled as an outpatient 3-h infusion, paclitaxel/cisplatin was moderately cost-effective at $30 619 per LYG. When compared with historical controls treated with best supportive care, this regimen of paclitaxel/cisplatin cost $4539 per LYG. Assuming a 3-h paclitaxel infusion yields the same survival advantage as the 24-h infusion did in the randomized trial, paclitaxel/cisplatin is a cost-effective improvement over standard etoposide/cisplatin for patients with advanced non-small cell lung cancer. © 1999 Cancer Research Campaign


British Journal of Cancer (1999) 80(5/6), 815–820
© 1999 Cancer Research Campaign
Article no. bjoc.1998.0426

### Cost-effectiveness of paclitaxel plus cisplatin in advanced non-small-cell lung cancer

**CC Earle and WK Evans**

Ottawa Regional Cancer Centre, University of Ottawa and Cancer Care Ontario, 501 Smyth Road, Ottawa, Ontario, Canada K1H 8L6

**Summary** The aim of this study was to assess the cost-effectiveness of combination chemotherapy with paclitaxel/cisplatin, compared with standard etoposide/cisplatin in patients with advanced non-small cell lung cancer (NSCLC). We obtained the primary survival and resource utilization data from a large three-arm randomized trial comparing: paclitaxel 135 mg $m^{-2}$ by 24-h intravenous (i.v.) infusion + cisplatin; paclitaxel 250 mg $m^{-2}$ by 24-h i.v. infusion + cisplatin + granulocyte colony-stimulating factor (G-CSF); and standard etoposide/cisplatin in patients with stage IIIb or IV NSCLC. We also modelled the regimens with paclitaxel 135 mg $m^{-2}$ + cisplatin administered as an outpatient by 3-h infusion, as clinical data suggest that this is equivalent to 24-h infusion. We collected costing data from the Ottawa Regional Cancer Centre and applied it to the resources consumed in the randomized trial. We integrated these data into the Statistics Canada POpulation HEalth Model (POHEM), which generated hypothetical cohorts of patients treated with each regimen. The POHEM model assigned diagnostic work-up, treatment, disease progression and survival characteristics to each individual in these cohorts and tabulated the costs associated with each. We did sensitivity analyses around the costs of chemotherapy and its administration, and the survival differences between the two regimens. All costs are in 1997 Canadian dollars ($1.00 Canadian ~ £0.39 sterling). The perspective is that of the Canadian health care system. In the trial, the two paclitaxel-containing arms had almost identical survival curves with a median survival of 9.7 months compared with 7.4 months for etoposide/cisplatin. As administered in the trial, paclitaxel/cisplatin cost $76 370 per life-year gained (LYG) and paclitaxel/cisplatin/G-CSF $138 578 per LYG relative to etoposide/cisplatin. However, when modelled as an outpatient 3-h infusion, paclitaxel/cisplatin was moderately cost-effective at $30 619 per LYG. When compared with historical controls treated with best supportive care, this regimen of paclitaxel/cisplatin cost $4539 per LYG. Assuming a 3-h paclitaxel infusion yields the same survival advantage as the 24-h infusion did in the randomized trial, paclitaxel/cisplatin is a cost-effective improvement over standard etoposide/cisplatin for patients with advanced non-small cell lung cancer.

**Keywords:** non-small cell lung cancer; costs; chemotherapy; paclitaxel; cisplatin

Lung cancer is the leading cause of cancer death in North America (Parker et al, 1996). The majority of cases are of non-small cell histology, and most present with locally advanced or metastatic disease. Although modest survival gains have been made with cisplatin-based combination chemotherapy (Non-small Cell Lung Cancer Collaborative Group, 1995), the treatment for patients with advanced non-small cell lung cancer (NSCLC) has been unsatisfactory (Steward and Dunlop, 1995). However, several new agents with encouraging response rates (Goss et al, 1996) and modest toxicity (Thatcher et al, 1995) are giving oncologists cause for optimism about improving treatment results.

The taxanes have shown impressive activity in a number of human cancers, including NSCLC (Rowinsky and Donehower, 1995). Both paclitaxel (Taxol®) and its semisynthetic analogue docetaxel have shown response rates above 20% in uncontrolled trials (Fossella et al, 1994; Francis et al, 1994). Paclitaxel is particularly interesting because of reported survival rates at 1 year of 40% (Chang et al, 1993; Murphy et al, 1993).

We have previously reported that single-agent paclitaxel may be a cost-effective therapy for stage IV NSCLC when compared with best supportive care (BSC) on the basis of phase II analyses (Earle and Evans, 1997). Recently, a three-arm phase III study by the Eastern Cooperative Oncology Group (ECOG) has compared standard cisplatin plus etoposide versus cisplatin plus paclitaxel at two different dose levels, with or without granulocyte colony-stimulating factor (G-CSF) (Bonomi et al, 1996, 1997). It found higher response rates and a statistically significant improvement in survival in the paclitaxel-containing arms compared to etoposide/cisplatin. There was also a trend towards improved 1-year survival.

However, in a time of increasing fiscal constraint, the cost of new interventions is a concern that can inhibit their adoption into routine practice. Knowledge of their effectiveness relative to cost can better inform resource allocation decisions. Therefore, we undertook this study to estimate the cost-effectiveness of paclitaxel plus cisplatin in advanced NSCLC management, relative to standard etoposide plus cisplatin.

### METHODS

### The ECOG 5592 trial

Our analysis is based on this three-arm randomized comparison of 599 patients with stage IIIb or IV NSCLC. The groups were treated with:

*Received 2 July 1998*
*Revised 30 November 1998*
*Accepted 4 December 1998*

*Correspondence to:* WK Evans

1. paclitaxel 135 mg m$^{-2}$ by 24-h intravenous (iv.) infusion + cisplatin 75 mg m$^{-2}$
2. paclitaxel 250 mg m$^{-2}$ by 24-h i.v. infusion + cisplatin also at 75 mg m$^{-2}$ + G-CSF
3. standard etoposide 100 mg m$^{-2}$ i.v. × 3 days + cisplatin 75 mg m$^{-2}$.

The response rates were found to be higher with the paclitaxel-containing regimens: 26% in the paclitaxel/cisplatin group and 31% in the paclitaxel/cisplatin/G-CSF group, versus 12% for the etoposide/cisplatin group. The two paclitaxel-containing arms had almost identical survival and were grouped together for survival analysis. This revealed a statistically significant improvement in the median survival, 9.7 months in the combined paclitaxel arms compared with 7.4 months for etoposide/cisplatin (log-rank $P$ = 0.049). Additionally, 39% of patients treated with paclitaxel/cisplatin and 40% of those treated with paclitaxel/cisplatin/G-CSF were alive at 1 year, compared to 32% of those receiving etoposide/cisplatin. However, this was not statistically significant.

Five of seven quality of life indices assessed during the trial did not differ among the three treatment arms. The remaining two domains favoured those treated with paclitaxel-containing regimens: lung cancer symptoms were significantly better in the paclitaxel-treated patients ($P$ = 0.027) and there was a trend towards improved emotional well-being ($P$ = 0.079).

### Determination of treatment costs

In order to assess treatment costs, we obtained resource utilization data from the ECOG randomized trial. To ascertain the total direct cost to the Canadian health care system for these treatments, we had to make a number of assumptions. We determined the average doses and number of treatment cycles from the pooled drug administration records of patients in the trial and assumed that each patient in our analysis received this same treatment. We assumed that the 24-h infusions required 1 day of hospitalization. However, we also modelled the effect of giving paclitaxel 135 mg m$^{-2}$ by a 3-h outpatient infusion as is the current practice at the Ottawa Regional Cancer Centre (ORCC). We assumed there was no drug wastage.

Hospitalization for complications occurred in 8.8% of etoposide/cisplatin cycles, compared to 7.4% for paclitaxel/cisplatin and 9.0% for paclitaxel/cisplatin/G-CSF. These hospitalizations were predominantly for haematologic toxicity. We calculated the average cost for such admissions through the Ottawa General Hospital Case Costing System. We obtained physician fees from the most recent Ontario Health Insurance Plan (OHIP) Schedule of Benefits. Because the investigations done in a clinical trial often do not reflect usual practice, we modelled the pretreatment blood work and imaging tests required prior to and during chemotherapy administration on those used in routine care at the ORCC. We determined the cost of these tests from the OHIP Schedule of Benefits. We assumed that test results were not duplicated as patients moved through the health care system.

The amount of time spent by nursing and pharmacy personnel involved in preparing and administering each type of chemotherapy was measured by the staff of the ORCC. We calculated the cost of personnel time by multiplying the amount of professional time expended by the 1997 hourly rates at the ORCC. Finally, nursing staff tracked and costed the actual supplies used in the preparation of paclitaxel. We extracted the ‘hotel’ costs of clinic visits from the BR 5 study and inflated them to 1997 dollars. We assumed the cost of terminal care hospitalization for patients receiving paclitaxel to be similar to that of patients receiving chemotherapy in the BR 5 study, as determined in the economic analysis of that trial (Jaakkimainen et al, 1990).

### Survival data

We obtained the raw survival data of patients in the ECOG randomized trial (Bonomi et al, 1996, 1997) from Bristol-Myers Squibb. We incorporated it into our model using a piecewise Weibull function (Figure 1) and determined the average survival gain. The Weibull function is a standard, flexible, parametric survival model commonly used by biostatisticians to model failure time data in cancer patients.

### The lung cancer costing model

Statistics Canada developed the lung cancer costing model as part of a larger project to simulate the health of Canadians. The

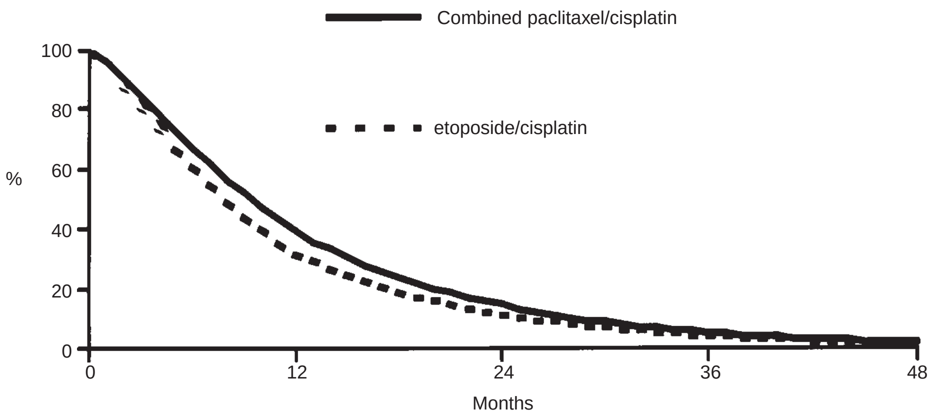

**Figure 1** Survival curves for the combined paclitaxel/cisplatin arms compared to etoposide/cisplatin in stage IIIb and IV non-small-cell lung cancer

 © Cancer Research Campaign 1999

POpulation HEalth Model (POHEM) is a software framework that integrates data on risk factors for major diseases, disease onset and outcome, health care utilization and direct care costs. The model generates a hypothetical cohort of people with demographic and labour force characteristics, risk factor exposures and health histories typical of Canadians. The perspective of the costing model is that of a provincial government payer in a universal health care system.

We have reported the lung cancer costing submodel previously (Evans et al, 1993, 1995*a*, 1995*b*, 1995*c*, 1997*a*, 1997*b*; Evans and Chevalier, 1996; Earle and Evans, 1997). In brief, it assigns individuals to a particular histologic cell type based on the distribution of these characteristics in the Canadian Cancer Registry. Stage distribution is based on retrospective chart reviews. It then assigns diagnostic work-up, treatment, disease progression and survival characteristics based on data from the medical literature, provincial registries and nationwide physician surveys. Finally, it allocates costs to the various components of care appropriate for cell type and stage of disease, from initial diagnosis through to terminal care. We assumed that terminal care costs were similar for patients in the three study arms. The model has recently been updated with 1992 incidence data. All costs are in 1997 Canadian dollars ($1.00 Canadian ~ £0.39 sterling). Because survival is very short for these patients, discounting was not applied.

We integrated the cost and survival data described above into POHEM to carry out our analyses. Cost-effectiveness, expressed as the cost per life-year gained (LYG) was calculated by the formula:

$$\text{Cost/LYG} = \frac{\text{cost}_1 - \text{cost}_2}{\text{survival}_1 - \text{survival}_2}$$

### Sensitivity analyses

Because clinical trials often produce efficacy results that are superior to those seen in routine practice, we did sensitivity analyses in which we decreased the survival differences between the regimens by 25 and 50%. A generic version of paclitaxel has recently become available in Canada, resulting in a decrease in price. Therefore, we did sensitivity analyses around the cost of chemotherapy and its administration, increasing it to pre-generic pricing. Because the majority of stage IV lung cancer patients in Canada are still managed without palliative chemotherapy (Raby et al, 1995), we also compared the survival of paclitaxel/cisplatin-treated patients to that of best supportive care (BSC). To do this, we modelled the survival of patients managed by BSC on the NCIC BR 5 trial (Jaakkimainen et al, 1990), a three-armed randomized trial comparing BSC to two chemotherapy regimens (Figure 2). We also did analyses restricted to stage IV patients only, as they did not benefit as much from chemotherapy as stage III patients in the ECOG 5592 trial.

### RESULTS

### Principal analysis

Table 1 presents a summary of the direct costs of chemotherapy administration for the different arms assessed in our model. In the ECOG 5592 study, patients in the etoposide/cisplatin arm received a median of four cycles of chemotherapy, as did those in the paclitaxel/cisplatin/G-CSF arm, while those in the paclitaxel/cisplatin arm received a median of five treatment cycles. The total cost of administering a course of paclitaxel (135 mg $m^{-2}$)cisplatin was $13 841 when given by 24-h infusion as an inpatient. This fell to $7832 when we modelled it at the same doses as an outpatient 3-h infusion. Paclitaxel, G-CSF and inpatient hospital care were the largest contributors to the cost of treatment.

The average survival of patients treated with paclitaxel/cisplatin calculated from the combined arms of the ECOG study exceeds that of etoposide/cisplatin by 1.6 months. From these data we were able to calculate that the paclitaxel/cisplatin arm as given in the trial costs $76 370 per LYG, while the paclitaxel/cisplatin/G-CSF regimen costs $138 578 per LYG. However, if the paclitaxel/cisplatin regimen could be given as an outpatient with the same effectiveness, the cost-effectiveness would improve to $30 619 per LYG (Table 2).

To put these numbers into a national perspective, in 1992 there were 4986 cases of stage IV NSCLC in Canada. The total cost to

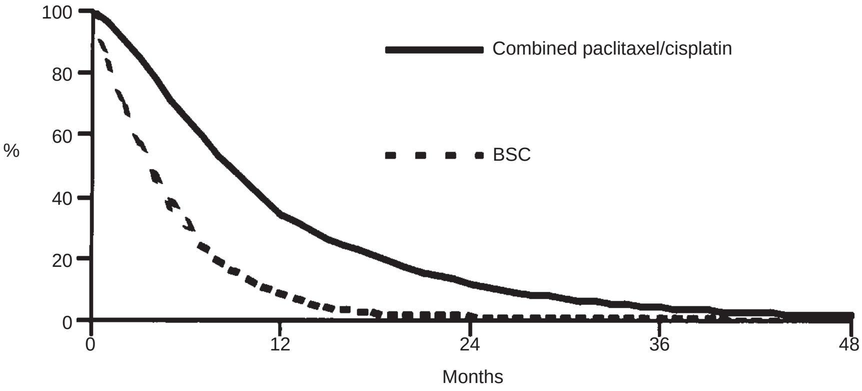

**Figure 2** Survival curves for the combined paclitaxel/cisplatin arms of the ECOG 5592 study compared to best supportive care arm of the NCIC BR-5 study in stage IV non-small-cell lung cancer

**Table 1** Summary of estimated treatment costs for the chemotherapy regimens in ECOG 5592 and ambulatory paclitaxel/cisplatin

| Item | Etoposide/cisplatin | Paclitaxel/cisplatin | Paclitaxel/cisplatin/G-CSF | Outpatient paclitaxel/cisplatin |
|---|---|---|---|---|
| Initial diagnosis and staging | $11 245 | $11 245 | $11 245 | $11 245 |
| Treatment costs | | | | |
| Number of cycles | 4 | 5 | 4 | 5 |
| Laboratory investigations | $366 (10%) | $458 (3%) | $573 (3%) | $458 (6%) |
| Drug costs | $1078 (30%) | $5755 (42%) | $15 017 (68%) | $5755 (73%) |
| Administration | $1241 (35%) | $6891 (50%) | $5512 (25%) | $882 (11%) |
| Toxicity | $876 (25%) | $737 (5%) | $896 (4%) | $737 (9%) |
| Total treatment costs (all cycles) | $3561 | $13 841 | $21 998 | $7832 |
| Terminal care[a] | $12 326 | $12 066 | $12 070 | $12 072 |
| Total costs | $27 132 | $37 152 | $45 313 | $31 149 |

[a]Terminal care costs attributable to lung cancer can vary due to differential lengths of survival and competing risks from other diseases.

**Table 2** Cost-effectiveness of various paclitaxel/cisplatin regimens compared to standard etoposide/cisplatin

| Regimen | Total cost | Incremental cost | Life-years gained | Cost/life-year gained |
|---|---|---|---|---|
| Etoposide/cisplatin | $27 132 | – | – | – |
| Paclitaxel/cisplatin (24-h infusion) | $37 152 | $10 020 | 0.1312 | $76 370 |
| Paclitaxel/cisplatin/G-CSF (24-h infusion) | $45 313 | $18 181 | 0.1312 | $138 578 |
| Outpatient paclitaxel/cisplatin (3-h infusion) | $31 149 | $4017 | 0.1312 | $30 619 |

Numbers may not add due to rounding.

**Table 3** Selected sensitivity analyses of paclitaxel/cisplatin compared to standard etoposide/cisplatin, with paclitaxel given by a 3-h outpatient infusion

| Manipulation | Cost per life-year gained |
|---|---|
| ↓ survival by 25% | $40 927 |
| ↓ survival by 50% | $71 321 |
| Stage IV patients only | $44 756 |
| Pre-generic paclitaxel costs | $49 028 |
| Compared to best supportive care | $4539 |
| Compared to best supportive care (stage IV only) | $5114 |

treat all of these with best supportive care would be $140 965 903. Treating them all with outpatient paclitaxel/cisplatin would cost $155 332 287, a difference of $15 366 384 (10%).

### Sensitivity analysis

Table 3 shows the effects of varying selected assumptions in the model. If the survival gain was only 50% of that reported, the cost-effectiveness ratio would rise to $71 321 per LYG. Considering only stage IV patients, who had less survival gain in the trial, the cost-effectiveness was still acceptable at $44 756. Using the higher cost of paclitaxel before it became generic, the cost-effectiveness ratio rose to $49 028. In non-randomized comparisons with BSC, paclitaxel/cisplatin cost $4539 per LYG for stage IIIb and IV patients, and $5114 per LYG if the analysis was restricted to stage IV patients.

### DISCUSSION

Our study suggests that the paclitaxel/cisplatin regimen can be a cost-effective improvement in the treatment of advanced lung cancer when given on an outpatient basis. Accepted thresholds for a cost-effective treatment intervention range from $20 000 to $100 000 per quality adjusted life-year (Laupacis et al., 1992). Most of our cost estimates fall within these guidelines.

High-dose paclitaxel given by 24-h inpatient infusion and supported with G-CSF was clearly not cost-effective when compared with etoposide/cisplatin. As has been observed in other situations (Canadian Coordinating Office for Health Technology Assessment, 1997), this strategy provided no advantage over lower dose treatment, but resulted in more toxicity. However, we found paclitaxel/cisplatin to be cost-effective when we modelled it given as a 3-h outpatient infusion. This assumes that the survival benefit would be similar despite this modification in its administration. Shorter paclitaxel infusions have been reported to be less toxic than longer infusions, with comparable response rates (Hainsworth et al, 1995). However, a recent randomized trial of 3- versus 24-h paclitaxel infusions in breast cancer found the longer infusion yielded a superior response rate (Mamounas et al, 1998). With respect to survival, two other randomized trials involving paclitaxel/cisplatin in advanced NSCLC found superior response rates but were unable to demonstrate a survival advantage for this regimen (Gatzemeier et al, 1998; Giaccone et al, 1998). Neither had standard control arms, so survival may have been similar in each trial because both regimens were superior to standard. However, both of these trials gave paclitaxel by 3-h infusion, raising the possibility that the shorter infusion duration decreased the survival benefit of treatment.

Our sensitivity analysis showed these results to be robust to most assumptions. When compared to best supportive care, the care most often given to advanced lung cancer patients in Canada (Raby et al, 1995), paclitaxel/cisplatin is a very cost-effective regimen. However, this analysis relied on a non-randomized comparison of survival experiences that may not accurately represent the survival benefit.

We did not directly incorporate quality of life adjustments into our analysis. In the clinical trial there was no significant difference in toxicity in any of the three arms. Furthermore, quality of life measures indicated that quality of life was as good or better in the

*© Cancer Research Campaign 1999*

paclitaxel-containing arms. As a result, calculation of costs per quality-adjusted life-year would not be expected to be significantly different from the costs per life-year gained in our analyses. If anything, improved quality of life would make paclitaxel/cisplatin more cost-effective.

Lung cancer is not an overly expensive disease to treat. However, by virtue of its high incidence it has a significant impact on total health care expenditures. Despite being cost-effective, treating all stage IV NSCLC patients in Canada with paclitaxel and cisplatin as outpatients would cost $155 million, an additional $15 million per annum compared to BSC. However, this is an overestimate because oncologists in Canada are still very conservative towards the treatment of advanced lung cancer, and would not offer treatment to all of their patients (Raby et al, 1995). In addition, many patients are not candidates for systemic therapy because of age, performance status, or co-morbid conditions. Consequently, the actual impact on health budgets of bringing paclitaxel/cisplatin into routine use is likely to be more modest. As advances in cancer research make more treatments available, society is increasingly asking practitioners to assess the costs and the benefits of the treatments provided. Given these considerations, outpatient paclitaxel/cisplatin chemotherapy can be considered both an effective and a cost-effective treatment for advanced NSCLC that is competitive with many other commonly accepted health care practices (Detsky and Naglie, 1990).

### ACKNOWLEDGEMENTS

The authors would like to thank the Health Analysis and Modelling Group at Statistics Canada (Jean-Marie Berthelot, B Phyllis Will, Christian Houle and Bill Flanagan) for their extensive help and support with the mathematical simulation, as well as for feedback on the manuscript. In addition, we would like to acknowledge the nursing and pharmacy staff at the Ottawa Regional Cancer Centre who helped collect costing data.

Dr. Earle is a Schering Fellow of the Canadian Society of Clinical Investigation. This study was supported in part by a grant from Bristol-Myers Squibb.

### Disclaimer

This analysis is based on Statistics Canada's POpulation HEalth Model of lung cancer management and cost. The assumptions and calculations underlying the results of the cost simulations were prepared by Drs Evans and Earle, and the responsibility for the use and interpretation of these data is entirely that of the authors.

### REFERENCES

Bonomi P, Kim K, Chang A and Johnson D (1996) Phase III trial comparing etoposide (E) cisplatin (C) versus taxol (T) with cisplatin-G-CSF (G) versus taxol-cisplatin in advanced non-small cell lung cancer. An Eastern Cooperative Oncology Group (ECOG) trial. *Proc Am Soc Clin Oncol* **15**: 1145 (Abstract)

Bonomi P, Kyungmann K, Kusler J and Johnson D (1997) Cisplatin/etoposide vs paclitaxel/cisplatin/G-CSF vs paclitaxel/cisplatin in non-small-cell lung cancer. *Oncology* **11**: 9–10

Canadian Coordinating Office for Health Technology Assessment (1997) *The Use of G-CSF in the Prevention of Febrile Neutropenia*. Canadian Coordinating Office for Health Technology Assessment (CCOHTA): Ottawa

Chang AY, Kim K, Glick J, Anderson T, Karp D and Johnson D (1993) Phase II study of Taxol, Merbarone, and Piroxantrone in stage IV non-small-cell lung cancer: the Eastern Cooperative Oncology Group results. *J Natl Cancer Inst* **85**: 388–394

Detsky A and Naglie I (1990) A clinician's guide to cost-effectiveness analysis. *Ann Int Med* **113**: 147–154

Earle CC and Evans WK (1997) A comparison of the cost of paclitaxel versus best supportive care in stage IV non-small cell lung cancer. *Cancer Prev Control* **1**: 282–288

Evans WK (1996) An estimate of the cost effectiveness of gemcitabine in stage IV non-small cell lung cancer. *Semin Oncol* **23**: 82–89

Evans WK and Chevalier T (1996) The cost-effectiveness of Navelbine alone or in combination with cisplatin in comparison to standard therapies in stage IV non-small cell lung cancer. *Eur J Cancer* **32A**: 2249–2255

Evans WK, Burpee C, Skinn B, Stewart DJ, Stapleton J, Armstrong J, Pollock D, Goss G and Logan D (1993) An evaluation of the costs of outpatient chemotherapy administration for small cell lung cancer. *Can J Oncol* **3**: 225–232

Evans WK, Will BP, Berthelot J-M and Wolfson MC (1995*a*) Estimating the cost of lung cancer diagnosis and treatment in Canada: the POHEM model. *Can J Oncology* **5**: 408–419

Evans WK, Will BP, Berthelot J-M and Wolfson MC (1995*b*) Diagnostic and therapeutic approaches to lung cancer in Canada and their costs. *Br J Cancer* **72**: 1270–1277

Evans WK, Will BP, Berthelot J-M and Wolfson MC (1995*c*) The cost of managing lung cancer in Canada. *Oncology* **9**: 147–153

Evans WK, Earle CC, Berthelot J-M, Will BP, Houle C and Flanagan B (1997*a*) The cost and cost-effectiveness of small cell lung cancer treatment in Canada. *Proc Am Soc Clin Oncol* **16**: 1503 (Abstract)

Evans WK, Will BP, Berthelot J-M and Earle CC (1997*b*) The cost of combined modality interventions for stage III non-small cell lung cancer. *J Clin Oncol* **15**: 3038–3048

Fossella F, Lee J and Murphy W (1994) Phase II study of docetaxel for recurrent or metastatic non-small cell lung cancer. *J Clin Oncol* **12**: 1238–1244

Francis P, Rigas J, Kris M, Pisters KMW, Orazem JP, Wooley KJ and Heelon RT (1994) Phase II trial of docetaxel in patients with stage III and IV non-small cell lung cancer. *J Clin Oncol* **12**: 1232–1237

Gatzemeier U, von Pawal J, Gottfried M, ten Velde GPM, Mattson K, DeMarinis F, Harper P, Salvati F, Robinet G and Lucenti A (1998) Phase III comparative study of high-dose cisplatin (HD-CIS) versus a combination of paclitaxel (TAX) and cisplatin (CIS) in patients with advanced non-small cell lung cancer (NSCLC). *Proc Am Soc Clin Oncol* **17**:

Giaccone G, Splinter TAW, Debruyne C, Kho GS, Lianes P, Van Zandwijk N, Pennucci MC, Scagliotti G, van Meerbeeckm J, van Hoesel Q, Curran DST and Postmus PE (1998) Randomized study of paclitaxel–cisplatin versus cisplatin–teniposide in patients with advanced non-small-cell lung cancer. *J Clin Oncol* **16**: 2133–2141

Goss GD, Dahrouge S and Lochrin CA (1996) Recent advances in the treatment of non-small cell lung cancer. *Anti-Cancer Drugs* **7**: 363–385.

Hainsworth J, Raefsky E, Thomas M and Greco F (1995) Paclitaxel administered by 1-hour infusion: Phase I/II study comparing two schedules of administration. *Proc Am Soc Clin Oncol* **14**: 376 (Abstract)

Jaakkimainen L, Goodwin PJ and Pater J (1990) Counting the costs of chemotherapy in a National Cancer Institute of Canada randomized trial in non-small cell lung cancer. *J Clin Oncol* **8**: 1301–1309

Laupacis A, Feeny D, Detsky, A and Tugwell P (1992) How attractive does a new technology have to be to warrant adoption and utilization? Tentative guidelines for using clinical and economic evaluations. *Can Med Assoc J* **146**: 473–481

Mamounas EP, Brown A, Smith RD, Lembersky B, Fisher B, Wickerham DL, Wolmark N, Atkins J, Shibata H and Baez L (1998) Effect of Taxol duration of infusion in advanced breast cancer (ABC): results from NSABP B-26 trial comparing 3- to 24-h infusion of high dose Taxol. *Proc Am Soc Clin Oncol* **17**: (Abstr 389)

Murphy WK, Fossella FW, Winn RJ, Shin DM, Hynes HE, Gross HM, Davilla E, Leimert J, Dhingra H, Raber MN, Krakoff IH and Hong WK (1993) Phase II study of Taxol in patients with untreated advanced non-small-cell lung cancer. *J Natl Cancer Inst* **85**: 384–388

Non-small Cell Lung Cancer Collaborative Group (1995) Chemotherapy in non-small cell lung cancer: a meta-analysis using updated data on individual patients from 52 randomised clinical trials. *Br Med J* **311**: 899–909

Parker S, Tong T, Bolden S and Wingo P (1996) Cancer Statistics, 1996. *CA Cancer J Clin* **65**: 5–27

Raby, B, Pater J and Mackillop W (1995) Does knowledge guide practice? Another look at the management of non-small-cell lung cancer. *J Clin Oncol* **13**: 1904–1911

Rowinsky EK and Donehower RC (1995) Drug therapy: paclitaxel (Taxol). *N Engl J Med* **332**: 1004–1014

Steward W and Dunlop D (1995) New drugs in the treatment of non-small cell lung cancer. *Ann Oncol* **6**: S49–S54

Thatcher N, Ranson M, Lee M, Niven R and Anderson H (1995) Chemotherapy in non-small cell lung cancer. *Ann Oncol* **6**: S83–S95

*© Cancer Research Campaign 1999*
